# Obesity in Prader–Willi syndrome: physiopathological mechanisms, nutritional and pharmacological approaches

**DOI:** 10.1007/s40618-021-01574-9

**Published:** 2021-04-23

**Authors:** G. Muscogiuri, L. Barrea, F. Faggiano, M. I. Maiorino, M. Parrillo, G. Pugliese, R. M. Ruggeri, E. Scarano, S. Savastano, A. Colao, RESTARE

**Affiliations:** 1 Sezione di Endocrinologia, Unità di Endocrinologia, Dipartimento di Medicina Clinica e Chirurgia, Università Federico II di Napoli, Via Sergio Pansini 5, 80131 Naples, Italy; 2 Cattedra Unesco “Educazione alla Salute e allo Sviluppo Sostenibile”, Università “Federico II” di Napoli, Naples, Italy; 3 Dipartimento di Scienze Umanistiche, Università Telematica Pegaso, Napoli, Italy; 4 Ambulatorio Diabetologia, Asp Cosenza, Cosenza, Italy; 5 Unit of Endocrinology and Metabolic Diseases, University of Campania “Luigi Vanvitelli”, Naples, Italy; 6 Endocrinology and Metabolic Disease, AORN S. Anna S. Sebastiano Caserta, Caserta, Italy; 7 Department of Clinical and Experimental Medicine, University of Messina, Messina, Italy

**Keywords:** Prader–Willi syndrome, Obesity, Hyperphagia, Diabetes mellitus

## Abstract

Prader–Willi syndrome (PWS) is a genetic disorder caused by the lack of expression of genes on the paternally inherited chromosome 15q11.2-q13 region. The three main genetic subtypes are represented by paternal 15q11-q13 deletion, maternal uniparental disomy 15, and imprinting defect. Clinical picture of PWS changes across life stages. The main clinical characteristics are represented by short stature, developmental delay, cognitive disability and behavioral diseases. Hypotonia and poor suck resulting in failure to thrive are typical of infancy. As the subjects with PWS age, clinical manifestations such as hyperphagia, temperature instability, high pain threshold, hypersomnia and multiple endocrine abnormalities including growth hormone and thyroid-stimulating hormone deficiencies, hypogonadism and central adrenal insufficiency due to hypothalamic dysfunction occur. Obesity and its complications are the most common causes of morbidity and mortality in PWS. Several mechanisms for the aetiology of obesity in PWS have been hypothesized, which include aberration in hypothalamic pathways of satiety control resulting in hyperphagia, disruption in hormones regulating appetite and satiety and reduced energy expenditure. However, despite the advancement in the research field of the genetic basis of obesity in PWS, there are contradictory data on the management. Although it is mandatory to adopt obesity strategy prevention from infancy, there is promising evidence regarding the management of obesity in adulthood with current obesity drugs along with lifestyle interventions, although the data are limited. Therefore, the current manuscript provides a review of the current evidence on obesity and PWS, covering physiopathological aspects, obesity-related complications and conservative management.

## Introduction

Prader–Willi syndrome (PWS) is a complex multisystem disorder that is caused by the lack of expression of paternally active genes in the PWS critical region on chromosome 15 (15q11.2-q13). Subjects with PWS are characterized by short stature, several endocrine diseases such as hypogonadism, growth hormone/insulin-like growth factor-I axis dysfunction, hypothyroidism, central adrenal insufficiency, dysmorphic features, scoliosis, osteoporosis, mental retardation, and behavioral and psychiatric diseases. One of the main characteristics of PWS is severe obesity, whose prevalence varies according to age. Indeed, the prevalence of overweight and obesity in PWS is around 40% in children and adolescents [35], while this percentage tends to increase ranging from 80 to 90% in adulthood [52, 104]. Although subjects with PWS have poor feeding and appetite in infancy, they developed uncontrolled appetite leading to weight gain after 3 years. It is noteworthy to observe that obesity in subjects with PWS has different characteristics than simple obesity [25]. Lean body mass is lower while fat mass is higher in subjects with PWS compared with subjects with simple obesity having similar body mass index (BMI) [14, 33]. Subjects with PWS showed lower visceral adiposity compared to subjects with simple obesity and this could account for the higher insulin sensitivity detected in subjects with PWS [33] and less prevalence of dyslipidemia [33]. Although subjects with PWS seem to develop less frequent cardiovascular risk factors than subjects with simply obesity, some case reports of cardiovascular events have been reported mostly at a young age [72, 89]. Although the milestone of the treatment of obesity in PWS is the prevention that could occur thanks to growth hormone (GH) therapy and lifestyle interventions started in childhood [32], nutritional interventions along with the use of current antiobesity drugs provide evidence to be effective in adult subjects with obesity [44]. Therefore, the current manuscript aims to provide an overview of the current evidence on obesity in PWS, starting from a physiopathological point of view to obesity-related complications and conservative management.

## Pathophysiology of obesity

**Fig. 1:**
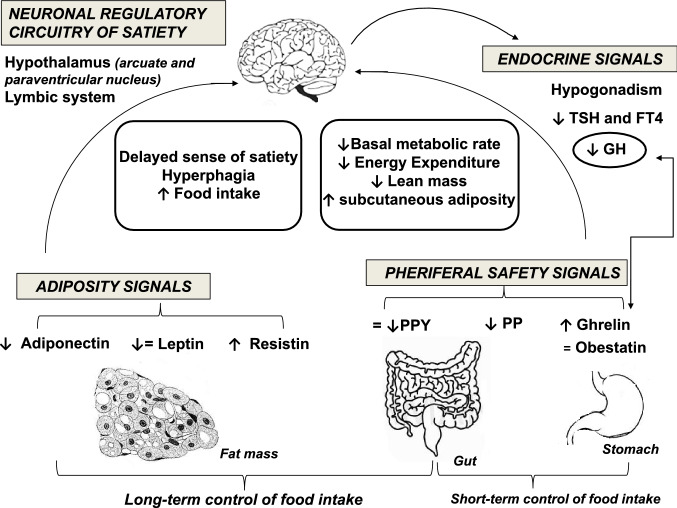
Mechanisms involved in the development of obesity in Prader–Willi syndrome (PWS). The proposed mechanisms include disruption in limbic-hypothalamic pathways of satiety control resulting in hyperphagia, alterations in hormones regulating food intake, reduced energy expenditure. Persistent increase in plasma ghrelin results in increased appetite via the central regulating mechanisms in the hypothalamus and increased food intake. Decreased plasma PP and PYY contribute to failure to satiety control. The role of leptin is still under investigation, as overall evidence suggests that leptin is unlikely to be misregulated in PWS obesity. Deficiency of GH and hypogonadism result in reduced muscle mass and increased body fat. Central hypothyroidism may contribute to reduced energy expenditure. Higher levels of ghrelin, adiponectin and resistin are associated with increased adiposity. *GH* growth hormone, *PYY* peptide YY, *PP* pancreatic polypeptide, *TSH* thyroid-stimulating hormone

Obesity represents a hallmark of the disease and a major cause of morbidity and mortality, along with its complications in PWS [82]. Obesity in subjects with PWS occurs through different mechanisms summarized in Fig. 1.

### Nutritional phases

Obesity develops starting from the second year of life, after an initial phase of poor feeding [79]. A gradual shift occurs over several nutritional phases through which subjects with PWS typically progress [25, 79]. Before birth, decreased fetal movements and growth restriction are observed with lower birth weight than siblings (phase 0). In early infancy (phase 1), hypotonia, poor suck and failure to thrive led to poor feeding and reduced appetite initially (0–9 months of age); from 9 to around 24 months of life, both feeding and appetite improve and weight increases at a normal rate. Starting from 2 years (phase 2), patients begin to gain weight due to a progressive increase in appetite and food interest. Hyperphagia, lack of satiety and obesity become prominent by school age (median age of onset: 8 years) and predominate during childhood (phase 3) through adulthood, although most but not all adults may develop satiety and resolution of food seeking (phase 4).

### Hypothalamic regulation of satiety

Despite the exact mechanisms remain to be fully elucidated, the development of obesity is mainly related to dysfunction in the hypothalamic satiety centre and its hormonal regulatory circuitry, affecting food intake and energy expenditure. Disruption in the hypothalamic pathways of satiety control results in persistent and insatiable appetite, hyperphagia and hunger-related eating behaviours. Aggressive and obsessive food seeking and storage, eating of inedibles, stealing of food or money to buy food are common among subjects with PWS, along with behaviour with features of autism spectrum disorder (controlling and manipulative behaviour, compulsivity, and difficulty with changes in routine), which favours abnormal eating [66, 79, 82].

Alterations in several brain areas (hypothalamus, amygdala, hippocampus, orbitofrontal and medial prefrontal cortex) play a crucial role in the abnormal food intake regulation in PWS. Functional magnetic resonance (MR) imaging demonstrated pre-meal higher activity in reward/limbic regions (nucleus accumbens, amygdala) and lower activity in the hypothalamus and hippocampus in response to food (vs non-food) in subjects with PWS compared to subjects with obesity non-PWS. Mostly, subjects with PWS exhibited a greater post-meal stimulation of food activation centres in the limbic and paralimbic region (hypothalamus, amygdala, hippocampus) and a lower activation in cortical inhibitory regions (orbitofrontal cortex, medial prefrontal cortex) [59, 60, 115]. This abnormal response is even higher for high-calorie vs. low-calorie foods [36]. Additionally, reduced functional connectivity between the ventral striatum and the limbic structures (hypothalamus and amygdala) was reported in subjects with PWS and it was correlated to obsessive eating behaviour [95]. These brain functional studies clearly indicate that disruption of hypothalamic control of satiety, dysfunction in the associated reward circuitry regions and impairment in inhibitory control areas jointly contribute to extreme hyperphagia and obesity in PWS.

### Alterations in hormones regulating food intake and adiposity

Several orexigenic and anorexigenic hormones are deemed to be involved in the development and maintenance of obesity in PWS, through appetite dysregulation.

- **Ghrelin**, a potent orexigenic hormone, is secreted by the stomach during fasting and starvation and its circulating levels are suppressed by food intake. Among the numerous physiological effects exerted, ghrelin increases appetite via central regulating mechanisms in the hypothalamus, stimulates GH secretion, regulates energy homeostasis and brown fat thermogenesis and also stimulates gastric emptying [67]. Most [17, 21, 34, 39, 69, 94, 96], but not all [37] studies reported persistently increased ghrelin levels in subjects with PWS at any age compared with BMI-, age- and sex-matched control children. Additionally, ghrelin levels remained elevated and did not appropriately suppress after eating in subjects with PWS compared to non-PWS controls with obesity [53]. The highest levels of ghrelin were found in the youngest children, even preceding the onset of increased appetite and obesity in PWS [45]. It has been hypothesized that the increase in circulating ghrelin levels occurs early in infancy, in response to difficulties to thrive and poor feeding. Persistent hyper-ghrelinemia promotes hyperphagia and obesity later in childhood [58], even if some Authors argued against a direct role of high ghrelin levels in the hyperphagia of PWS [69]. Recently, it has been demonstrated in mice that neonatal ghrelin programs the development of hypothalamic feeding circuits. Indeed, early postnatal chronic administration of ghrelin impairs the normal development of the hypothalamus, causes metabolic dysfunction and predisposes to obesity later in life [107]*.* In 2016, Beauloye et al. demonstrated that subjects with PWS in early infancy had normal acylated ghrelin (AG) levels, but high unacylated ghrelin (UAG) levels, confirming that total ghrelin is elevated at this age. Such an increase in total ghrelin was mainly due to the inactive form UAG, thus explaining poor appetite in early infancy, as opposed to that found later in life, when subjects with PWS display obesity and hyperphagia [12]. A subsequent study found that circulating ghrelin levels were elevated in children and adult subjects with PWS compared to both lean and obese controls as a result of increased active AG, whereas UAG was relatively low. The obese controls displayed the same relative deficit of UAG, but had lower levels of total ghrelin and lower AG/UAG compared to the lean controls [68]. Tauber and coworkers, who published the first study demonstrating that hyperghrelinaemia starts early in life, prior to hyperphagia and obesity [39], concluded for an impaired development of the ghrelin system in subjects with PWS, starting with excessive UAG, which then decreases, followed by an excessive increase in AG and a relative deficit in UAG. Subsequently, the AG/UAG ratio in subjects with PWS was significantly higher than in controls when the patients displayed hyperphagia, indicating an increase of this AG/UAG ratio throughout the nutritional phases. The ghrelin system is differently impaired at different ages and this impaired switch in the ghrelin system would explain the early excessive weight gain and subsequent obesity with hyperphagia observed in PWS [111, 112]***. ***However, several groups have shown that pharmacological reduction of ghrelin to normal levels in PWS, using either short or long-acting somatostatin analogue (SSA), did not improve feeding behaviour and did not reduce the appetite of children and adults with PWS nor affect the weight [55, 112]. Since the anorexigenic circuits related to ghrelin are disrupted early in life in PWS, it is conceivable that decreasing the level of ghrelin is not sufficient to reset the system in child, adolescents and adults. Finally, independent of its orexigenic effects, ghrelin stimulates adipose tissue lipogenesis and inhibits lipolysis, maybe contributing to increased adiposity in PWS [91].
- **Obestatin** is produced in the stomach by post-translational modification of ghrelin. In contrast to ghrelin, obestatin suppresses food intake, inhibits gastric emptying and decreases weight gain [114]. No significant difference has been reported in plasma obestatin levels between PWS and non-PWS subjects with obesity [90].
- **Pancreatic polypeptide** (PP) and **peptide YY** (PYY) are anorexigenic hormones released by the intestine post-prandially to induce satiety and inhibit eating [105]. PP levels were reduced in children with PWS [45]*,* while PYY levels were found to be either reduced [21] or increased [62] in subjects with PWS compared to either obese or not obese controls. Mostly, post-prandial PYY levels were similar in children with PWS and controls [17]. In addition, no differences between subjects with or without PWS emerged in the pattern of expression of ghrelin and PYY genes and their receptors in the brain [109].
- **Leptin** is a peptide produced by adipose tissue and is involved in the regulation of appetite and fat storage. Released by adipocytes in response to satiety signals, it reduces food intake and energy metabolism by inhibiting neuropeptide Y (NPY) neurons in the arcuate nucleus [92]. In the study by Goldstone et al., fasting leptin levels were significantly higher in subjects with PWS (*n* = 42, aged 7 months–5 years) compared to age-, sex- and BMI-matched controls (*n* = 9)*,* without any significant relationship with age, BMI and eating behaviour [45]*.* However, most studies failed to find any difference in leptin levels between subjects with or without PWS and obesity, as leptin levels were consistent with increased adiposity associated with obesity irrespective of aetiology [22, 47, 73, 85]*.* Thus, leptin is unlike to be misregulated in PWS.
- **Adiponectin** is another peptide produced by adipose tissue and plays a role in regulating adiposity. Serum adiponectin levels were found to be significantly lower in subjects with PWS compared with lean ones and significantly higher compared with obese controls [16]. Adiponectin is also associated with increased insulin sensitivity in subjects with PWS [54]. Recent evidence indicates that subjects with PWS are less likely to develop diabetes than non-PWS ones with comparable BMI [49]. This may be due to the altered fat distribution in subjects with PWS, resulting in greater adiponectin production than comparison subjects.
- Serum **resistin** and resistin mRNA expression in adipose tissue was significantly higher in subjects with PWS, compared with both healthy lean controls and subjects with obesity and without PWS. Moreover, higher levels of resistin were associated with lipogenesis in subjects with PWS and obesity, whereas no significant association was found between resistin and insulin resistance [88].

### Endocrine dysfunctions

Multiple endocrine abnormalities due to hypothalamic dysfunction are quite common in subjects with PWS and may represent factors contributing to abnormal body weight and composition. Growth hormone deficiency (GHD) is associated with decreased lean mass, increased fat mass (mainly truncal fat with increased waist/hip ratio), poor muscle tone and strength, decreased movements and reduced energy expenditure and exercise tolerance. In both children and adults with PWS, GH treatment reduced BMI and improved body composition, increasing lean mass and reducing fat mass. Also, muscle tone, exercise endurance and energy expenditure improved with GH treatment [8, 71, 106]. Moreover, studies following patients for 12–24 months after the cessation of GH replacement have shown a progressive increase in BMI and a tendency towards an increase in visceral adipose tissue [86]. Hypogonadism, occurring in almost 100% of subjects with PWS, is also associated with increased BMI and abnormal body composition (reduced muscle mass and increased fat mass) [82]. Similarly, the peripheral effects of thyroid hormone deficiency affect body composition, contributing to reducing energy expenditure. Indeed, subjects with PWS exhibit a lower amount of lean body mass and a higher level of fat mass compared to obese subjects with similar BMI [74]. The decrease in muscle mass is the result of endocrine dysfunctions (GH deficit and hypogonadism), severe muscle hypotonia, lower physical activity, behavioral issues and reduced both resting and activity energy expenditure (EE), despite a normal relationship between fat-free mass and EE [14]. The distribution of fat and lean mass differ between body sites (e.g., between lumber and spine areas and the hips and thighs). The excessive fat mass in subjects with PWS is typically distributed to the trunk and to the proximal extremity of the limbs, with a lower trunk-to-appendicular fat mass ratio and prevalent subcutaneous and less visceral fat accumulation. According to the reduction in visceral adiposity, preserved insulin sensitivity and lower metabolic consequences are observed in subjects with PWS in comparison to BMI-matched obese controls [18, 48, 82, 108]. Possible explanations could be the prevalent subcutaneous and less visceral fat accumulation, higher ghrelin and adiponectin levels as well as lower GH hormone levels [48, 82].

## Obesity-related comorbidities

### Type 2 diabetes mellitus

PWS is often complicated by glucose intolerance and the development of diabetes mellitus. The reported prevalence of diabetes in subjects with PWS ranges from 7 to 24%, with higher rates in obese adults after the 5th decade and lower rates in prepubertal age [35, 104]. Moreover, a retrospective study revealed an earlier onset of diabetes (median age of 15 years) in 65 Japanese subjects with PWS, maybe due to the higher rate of metabolic disorders in Asian diabetic children than in Caucasians [113]. The most frequent form of diabetes in PWS patients is represented by type 2 diabetes mellitus (T2DM) [35, 104, 113]. Obesity and weight gain are well-known risk factors for the development of insulin resistance and T2DM [38]. However, the role of overweight/obesity in the pathogenesis of T2DM in subjects with PWS remains controversial. Both cross-sectional [13] and longitudinal [110] studies showed that the distribution of fat in subjects with PWS is predominantly subcutaneous, as compared with individuals with non-syndromic obesity, with lower levels of insulin resistance. Moreover, subjects with PWS showed lower fasting insulin levels despite severe obesity grade [70]. In this study, Lacroix et al. compared metabolic profile and fat distribution in 42 PWS adults with matched subjects with primary obesity reporting that the PWS group had a lower percentage of trunk fat mass and a better metabolic profile, in particular a lower insulin resistance assessed by HOMA index than the control group. It has been widely demonstrated that PWS subjects showed a state of relative hypoinsulinemia, without the expected insulin resistance, despite their severe obesity. The reasons for the lacking onset of insulin resistance sensitivity in PWS patients could be due to a small amount of visceral fat compared to total adiposity, an impaired GH secretion and higher levels of ghrelin considering the degree of obesity [30]. At the early onset of T2DM, subjects with PWS are generally asymptomatic or may present the classic Hippocrates' triad of diabetic symptoms (polyuria, polydipsia and unexpected weight loss) [11]. Periodic and regular evaluation of glucose levels, glycosylated hemoglobin (HbA1c), lipid profile and blood pressure should be recommended in all subjects with PWS since childhood [30]. An oral glucose tolerance test (OGTT) should be performed in subjects with PWS and obesity at pubertal and adult age. Moreover, both glucose and insulin levels should always be evaluated before and after starting GH therapy, as GH has counter-regulatory effects on glucose metabolism [30]. The diagnostic criteria for diabetes and prediabetes (i.e., impaired fasting glucose) to be followed for subjects with PWS are the same used for the general population [7]. In the long-term, subjects with PWS and T2DM seem to be less prone to develop diabetic-related complications as compared to general diabetic patients [11]. However, at the time of diagnosis of T2DM and thereafter, screening for microvascular complications (retinopathy, nephropathy and neuropathy), hypertension and cardiovascular disease should be carried out annually. The primary goal of the treatment of T2DM in subjects with PWS is to improve the quality of life, while reducing weight and achieving an optimal glycaemic control. The first-line therapy is based on a restriction of caloric intake and regular exercise to promote weight loss and improve glycemic control [76]. Pharmacological strategies for T2DM in subjects with PWS do not differ from those followed for the general population [6]. Metformin represents the first-line glucose-lowering agent to choose in combination with lifestyle modification since it helps to reduce satiety and improve insulin sensitivity, promoting weight loss. Alpha-glucosidase inhibitor, acarbose, may be a promising alternative option. It is associated with reduction of body weight, post-prandial glucose levels and daily insulin doses. In a study on Japanese population, alpha-glucosidase inhibitors were used by more than 50% of subjects with PWS and T2DM [113]. On the other hand, thiazolidinediones, sulfonylureas and insulin regimens are not recommended due to weight gain and risk of hypoglycemia. Glucagon-like peptide-1 receptor agonists (GLP-1RAs) and dipeptidyl peptidase-4 (DPP-4) inhibitors represent both valid and effective options to enhance weight management and HbA1c control in subjects with PWS [44, 56]. Data regarding the recent and innovative Sodium-glucose co-transporter-2 (SGLT2) inhibitors are rather scarce. Use of canagliflozin on a 40-years-old diabetic woman with PWS resulted in an improvement of HbA1c and in a decrease of body weight [65]. Currently, the evidence regarding the combined use of GLP-1 agonists and SGLT-2 inhibitors in the treatment of type 2 diabetes mellitus in PWS is limited to some case reports. In a 20-year-old woman with PWS, the addition of SGLT2 inhibitor, empagliflozin (10 mg/day) to existing liraglutide therapy resulted in significant improvements in both weight and glycemic control: a weight loss of approximately 5.5 kg (7.4%) was achieved during the subsequent 5 months without altering dietary intake and HbA1c level notably improved from 9.2 to 7.2% [101]. In an adolescent with T2DM and PWS the add-on of Metformin to different insulin regimens or Liraglutide did not improve glucose levels. Liraglutide and Empaglifozin (SGLT-2 inhibitor) therapy used in combination were well tolerated and rapidly normalized blood glucose and HbA1c (48 mmol/mol) which was sustained after 6 months of treatment [23]. Insulin therapy should be considered when oral glucose-lowering agents are no longer effective in glycaemic control or when there is evidence of insulin deficiency (ketoacidosis and unexplained weight loss). Moreover, in case of T2DM with serious obesity morbidities, bariatric surgery should be an alternative worth considering to achieve a rapid and beneficial weight loss [30]. It helps to reduce ghrelin levels and increase Glucagon-like peptide 1 (GLP-1) secretion, even if long-term effects on weight loss are less effective than in non-syndromic obesity. It should be considered only in selected subjects with PWS on psychological support when other therapeutic strategies have failed or are not available.

### Dyslipidemia

Few studies investigated dyslipidemia in subjects with PWS [20, 48, 54, 108]. Butler et al. [20] enrolled 26 subjects with PWS (14 males/12 females, mean age 18.9 ± 7.0 years) and 32 subjects with obesity (6 males/26 females age 23.6 ± 6.6 years). No significant differences were found in terms of triglycerides (96.2. ± 67.0 vs 97.3 ± 61.8 mg/dl), total cholesterol (180.6 ± 46.5 vs196.1 ± 53.9 mg/dl), high-density lipoprotein (HDL) cholesterol (42.7 ± 12.9 vs 45.7 ± 14.2 mg/dl) and Low-density lipoprotein (LDL) cholesterol (136.1 ± 32.6 vs 141.4 ± 62.1 mg/dl) levels between subjects with PWS and subjects with obesity, respectively. Thus, the authors conclude that plasma lipid levels are not altered in subjects with PWS. Similar findings were found by Haqq et al. [54] that assessed lipid profile in 14 subjects with PWS (median age and BMI *Z* score: 11.4 and 2.15 years, respectively), 14 age and BMI matched children with obesity (median age and BMI Z score 12.0 and 2.35 years, respectively) and 14 age-matched lean children (median age and BMI Z score 12.3 and − 0.6 years, respectively). Fasting plasma total and LDL cholesterol were comparable between the three groups. As expected, children with obesity showed 38% lower HDL cholesterol levels (*p* < 0.001) and twofold higher plasma triglycerides (*p* < 0.001) compared to lean children. No significant differences were detected between children with PWS and lean children, while they showed significantly higher HDL and lower triglycerides compared to children with obesity [54]. Talebizadeh and Butler [108] compared 37 subjects with PWS (age 22.7 ± 9.5 years, BMI 36.5 ± 4.3 kg/m^2^) with 18 subjects with obesity (age 25.9 ± 13.3 years, BMI: 38.1 ± 5.8 kg/m^2^). No statistical differences were detected in plasma cholesterol, whereas fasting triglycerides were lower in the PWS group (158.4 ± 90.3 vs 252.4 ± 186.7 mg/dl). In this study, the authors also evaluate regional fat distribution using T1 weighted MR images and grouped subjects according to intra-abdominal visceral fat area (VFA) higher or less than 130 cm^2^. In the control group with obesity, no significant differences in plasma lipids were detected in the subjects with VFA ≥ 130 cm^2^ compared to the subjects with VFA < 130 cm^2^. However, in PWS group with high intra-abdominal VFA (VFA > 130 cm^2^) fasting triglycerides were significantly increased compared with the subjects with PWS with VFA < 130 cm^2^, but no differences in total cholesterol were found. These data suggest that in subjects with PWS adiposity localized in the abdominal region, visceral fat compared with subcutaneous fat, which is associated with high triglyceride plasma levels [108]. In conclusion, although the studies on dyslipidemia in PWS are few, most of them agree that subjects with PWS tend to have a lipid profile similar to lean subjects. This could be explained by the fact that subjects with PWS have less visceral fat content compared to BMI-matched subjects with obesity and this could preserve the lipid profile by the detrimental effect of fat in the context of this syndrome.

### Cardiovascular disease

Due to the multiple presence of cardiovascular risk factors such as obesity, hypertension, T2DM, syndrome of sleep apnea (OSAS), and low-grade chronic inflammation [9, 63] subjects with PWS are expected to be at risk of developing cardiovascular diseases. A nationwide cohort study carried out in Denmark in 155 subjects with PWS that were followed-up from birth through to the first occurrence of an outcome of interest found an increased risk of myocardial infarction (RR: 7.2; 95% CI 1.7–30.2) in these subjects compared to the general population [57]. However, cardiovascular diseases in PWS have been mostly detected in young age as reported in a 28-year-old woman with PWS that developed chest pain and loss of anterior R wave amplitude on the electrocardiogram [89]. A severe proximal stenosis of the left anterior descending artery with delayed antegrade flow together with antero-apical akinesia consistent with myocardial infarction has been detected at cardiac catheterization [89]. Also, an inferior wall myocardial infarction has been reported in 26-year-old white male with PWS and T2DM [72]. A graded exercise test using the Bruce protocol showed inferolateral ischemia. A subsequent cardiac catheterization showed severe, inoperable three-vessel coronary artery disease [72]. Although few cases of cardiovascular diseases have been reported in subjects with PWS, a screening and eventual treatment of cardiovascular risk factors in these patients is mandatory, mostly because cardiovascular events have been reported at a young age.

## Obesity prevention in PWS

Obesity is one of the principal features of PWS, which is linked to morbidities (including T2DM, metabolic syndrome, OSAS, respiratory insufficiency, and cardiovascular disease) and premature mortality [31]. Thus, it is important to prevent obesity in the first years of life and to implement strategies that allow the control of caloric intake. Unfortunately, in subjects with PWS this is not very easy because they have low compliance to follow food restrictions and they tend to consume any type of food, even unconventional or with bad taste, to satisfy their insatiability. Therefore, the obesity prevention approach must be carried out at several levels with dietary, physical and behavior interventions. It is advisable to consult a nutritionist who can establish a specific program characterized by a correct caloric intake in the first months [31] of life followed by a low-calorie, well-balanced diet and close supervision to minimize food stealing [27, 76]. Physical activity and muscle force training need to be an important part of daily life in subjects with PWS of all ages. It has been demonstrated that young subjects with PWS engaged in less overall physical activity than subjects with obesity without PWS, on both weekends and throughout the week [26]. Probably this is due to the fact that subjects with PWS have less muscle mass and lower muscle tone than other children [26]. Therefore, interventions should integrate activities of different intensity, to motivate patients to follow exercise programs [97]. Moreover, it is recommended to start these programs in young infants, to provide skill acquisition and strength training to increase motor development [97]. It is necessary to educate subjects with PWS to manage anxiety and follow the recommended programs. To adhere to all the suggested recommendations (prescribed diet, eating routines, healthy eating behaviors, daily exercise), patients need to be supported by behavioral programs in which are included teachers, friends and family members [50]. At last, to prevent the development of obesity, GH treatment could have an important role. In subjects with PWS, GH has beneficial effects on body composition, basal energy consumption, muscle strength, exercise tolerance and decrease in free fat mass [24, 41, 80, 84] and many studies revealed that effects on body composition are greater in the first year of treatment [33, 35, 43]. In fact, one of the studies in which subjects with PWS received therapy for a long period [8] starting at the age of 3–7 years, reported that lean body mass significantly increased and fat SD% significantly decreased in the first year of treatment, while no significant difference was observed in the subsequent years and at the end of the 8-year treatment period. Regarding BMI, values in PWS remained higher than normal children but were below that of non-treated children with PWS, thus suggesting that GH therapy could have a role in hindering obesity. However, it should be pointed out that response to GH therapy changes according to age of starting treatment. In a study that compared subjects with PWS receiving GH therapy (starting age 4–20 months) with those who did not, it was reported that the treated group had a lower body fat, increased muscle mass, better lipid profile and better motor function [24], thus suggesting that early treatment might explain the significant differences observed.

## Nutritional management of obesity in PWS

Nutritionists play a major role in the management of obesity in PWS, considering that dietary restriction is one of the only successful treatments in the reduction and maintenance of body weight in this disease. Whereby, nutritionists as integral members of an interdisciplinary team must understand the complexity of this disease. Infants with PWS at birth have a normal length and are either normal weight or underweight [14, 46]. However, the presence of hypotonia, which also affects the oral cavity, commonly leads to a poor sucking ability. This leads to failure to thrive, which represents the first stage of PWS throughout infancy. The nutritional goal for infants with PWS is to promote proper growth without leading to overweight or obesity. To do this, the infants with PWS should be kept between the 50th and 75th percentile of his/her weight for height [98]. Of interest, the basal metabolic rate, the minimum energy required to maintain vital body functions [93], in infants with PWS is slower than an infant without PWS, suggesting that infants with PWS require fewer calories than the healthy counterparts. In preschool and school-age children with PWS the guidelines recommend from 10 to 11 cal per centimeter of length to keep the weight and 8 to 9 cal per centimeter to reduce body weight [98]. For young children, the guidelines recommend from 600 to 800 cal per day while for older children and adults from 800 to 1100 cal per day [19]. In addition, it is important in subjects with PWS to take a multivitamin, and above all take a vitamin D supplement if necessary, to compensate for the reduced intake of vitamins and minerals that may be suboptimal due to a restrictive diet [10, 98]. When subjects with PWS develop obesity in adulthood, body weight should be assessed frequently and the total energy intake adjusted accordingly.

Currently, the best nutritional strategy in PWS subjects would appear a well-balanced low-calorie diet, in fact, in a study on 63 subjects with PWS, it has been reported that a balanced energy-restricted diet of approximately 30% fat, 45% carbohydrates (at least 20 g of fiber per day) and 25% protein, significantly improved both weight and body composition compared to a simple energy-restricted diet [78]. Some studies have evaluated the effect of low-carb diets in PWS subjects. In a clinical study, the efficacy and safety of the modified Atkins Diet for 4 months (low carbohydrate and high fat) was assessed in seven children with PWS ages 6–12 years who were overweight/obese; it was shown that only one patient lost 2.9 kg while the others did not change their weight. Positive effects on hyperphagia and behavior were subjectively reported by families [40]. Another study was carried out by Irizarry et al. to investigate the effect of different diets on hormonal and metabolic balance in PWS: eight PWS children (age 9–18 years) were randomized to consume either low-carbohydrate, high-fat (LC, 15% carb; 65% fat; 20% protein) or low-fat, high-carbohydrate (LF, 65% carb, 15% fat, 20% protein) diets matched for calories and protein, during a first hospital admission and the second nutritional pattern during a subsequent admission, collecting blood samples after overnight fasting and 1 h after a mixed meal. It was observed that subjects consuming the LC diet had lower postprandial insulin concentrations, higher fasting GLP-1 and GIP concentrations, increased postprandial GLP-1 and reduced ratio of fasting ghrelin to GLP-1 compared to LF diet. Although further studies on a larger sample are needed, this evidence suggests that increases in GLP-1 with LC feeding and reductions in the ratio of ghrelin to GLP-1 might limit food intake and improve glycaemic control in PWS [64].

## Pharmacological treatment of obesity in PWS

As extensively illustrated above, obesity in the subject with PWS seems to depend on pathophysiological mechanisms that are in part different from idiopathic obesity and, therefore, also regarding pharmacological therapy, it could benefit from some categories of drugs with mechanisms of action various and innovative. Metformin is an oral hypoglycaemic drug indicated in T2DM, but also used off-label in conditions of obesity and prediabetes [5]. Its mechanism of action is not fully known, although, in addition to the known insulin-sensitizing effect on the liver and muscle, it also appears to have an anorectic effect: central to the hypothalamic-pituitary circuits and peripheral due to increased secretion of GLP-1 from the intestine [75]. In a pilot study of 21 children and adolescents with PWS with insulin resistance and glucose intolerance on OGTT that started for this reason treatment with metformin, it has been observed an improvement of food-related distress and anxiety, evaluated by Hyperphagia Questionnaire (HQ) however without a reduction in body weight [77]. In addition, 7 out of 10 males had to stop metformin after 1–2 days for a marked deterioration in behavior.

Naltrexone–bupropion combines drugs already tested for some decades in monotherapy and already in use for the treatment of alcohol and opioid dependence (Naltrexone), and major depression or smoking cessation (Bupropion). This combination determines a pharmacological hypothalamic synergistic mechanism in suppressing appetite and lowering body weight [51]. As for the mechanism of action, Bupropion is a mild inhibitor of dopamine and norepinephrine reuptake, which promotes the conversion of pro-opiomelanocortin (POMC) into α-melanocyte-stimulating hormone (α-MSH), while Naltrexone prolongs its duration of action through retro-regulation of the μ opioid receptor, therefore Naltrexone/bupropion acts synergistically to activate POMC neurons in the hypothalamic, in particular in the neurons in the arcuate nucleus (ARC), resulting in appetite suppression [1]. There are two case reports on the use of naltrexone alone on a child and four adolescents, respectively [15, 116]: the first showed after 4 months of therapy a weak improvement in weight control and behavior, while the second, in which the treatment lasted one week, did not demonstrate a reduction in appetite, at least in the short term. The only described case of combination therapy with naltrexone/bupropion for 6 months showed an improvement of eating habits, without a significant reduction of BMI in an adolescent girl with PWS [61].

GLP-1RAs, such as exenatide and liraglutide, act by stimulating glucose-dependent insulin secretion and determining a decrease in appetite and weight, with protective effects on pancreatic β-cells and cardiovascular system, representing an effective drug for diabetes and obesity [28]. In a report of 6 cases of diabetic subjects with PWS treated with 1.2 to 1.8 mg/day of liraglutide (4 patients) and 20 mg/day of exenatide (2 patients), during the 24 months of treatment a tendency to decrease BMI, HbA1c, waist circumference and mean glycaemia was detected [44]. In a longitudinal study, the efficacy of exenatide therapy for 6 months in overweight or obese young adults with PWS was assessed and a reduction in appetite and an improvement in HbA1c without change in weight or BMI was observed [100]. Until now, no data are available on the use of dulaglutide or semaglutide in subjects with PWS which could be particularly useful in weight control.

Orlistat is a pancreatic lipase inhibitor useful for limiting fat absorption to up to 30% of ingested fat and approved for the treatment of obesity [99], however, studies evaluating the efficacy and safety in subjects with PWS are lacking. Moreover, regarding lorcaserin, a high-affinity selective agonist of the serotonin 2C receptor with a weak efficacy on weight loss [42], studies on subjects with PWS have not been performed. Moreover, in January 2020, Food and Drug Administration (FDA) requests the withdrawal of lorcaserin from the market because a safety clinical trial shows a possible increased risk of cancer during treatment with this drug. Two different drugs: sibutramine, an unspecific inhibitor of serotonin and norepinephrine reuptake, and the rimonabant, an endocannabinoid CB1 receptor antagonist, which act by reducing appetite and increasing energy expenditure, while appearing promising in subjects with PWS, have been withdrawn due to serious cardiovascular and psychiatric side effects, respectively, that emerged during clinical trials [81, 87].

Topiramate is an antiepileptic drug that acts as a modulator of Na+ channels, GABA, and AMPA/kainate receptors, it is useful in the treatment of atypical psychoses of subjects with PWS and it is also known to affect food-seeking behavior [4]. A double-blind randomised placebo-controlled 8-weeks trial was conducted on 62 subjects with PWS to study the efficacy and tolerance of the topiramate on behavioural disorders and it was observed that behaviour and severity scores evaluated by Dykens Hyperphagia Questionnaire (DHK) improved significantly more over time in topiramate group versus placebo group, with a significant dose–effect relationship, however, without a significant reduction of BMI [29].

Based on the numerous pathophysiological mechanisms proposed for obesity in PWS, there are currently several ongoing randomized clinical trials on different drugs such as diazoxide, beloranib, setmelanotide, intranasal oxytocin and oxytocin analogs, carbetocin and livoletide [31]. These molecules have different targets mostly acting on lipid metabolism and on the central circuits of hunger and satiety. In particular, livoletide, an unacylated ghrelin analog, after having demonstrated in a phase 2 randomized clinical trial a significant improvement in food-related behaviors [2], supported by a reduction in hunger, is currently under study in a phase 3 clinical trial. In the context of drugs being tested for the control of hyperphagia in PWS, the association of tesofensine (a presynaptic absorption inhibitor of noradrenaline, dopamine and serotonin) and metoprolol (a selective beta-blocker) is currently in phase 2b of experimentation and has already shown promising results, in fact, in a phase 2a study involving 18 patients with PWS, this association reduced body weight, improved BMI and reduced hyperphagia to very low levels.

## Bariatric surgery in PWS

Despite the number of cases of PWS subjects undergoing bariatric surgery did not report encouraging results in terms of weight loss and development of complications [103], more recent studies in which obsolete surgical techniques were avoided and mainly laparoscopic sleeve-gastrectomy (SG) and mini gastric bypass (MGB) were used [3, 83, 102], have shown that bariatric surgery can be a useful tool in the management of weight excess in selected cases of patients with PWS, with positive results in terms of weight loss and a reduced number of complications. One of the most controversial aspects remains PWS patients’ compliance in the post-surgical frame time. Indeed, in the general population, a psychiatric assessment is carried out before bariatric surgery to investigate if the very same subjects are able to follow post-surgical indications to guarantee a result in terms of adequate weight loss and reduce the incidence of complications. Although most of the time PWS patients did not show a promising post-surgical compliance, bariatric surgery is the only possibility for weight loss A study on 60 American patients with PWS showed after 5 years from surgery a weight loss of only 2.4%, significantly less than that observed in non-PWS subjects with obesity, however, as anticipated this data could be influenced by different types of surgery performed, some of which are now obsolete (54% biliopancreatic diversion, 29% gastric bypass, 18% bioenteric intragastric balloon, 5.4% jejunoileal bypass, 3.6% gastroplasty, 3.6% vertical banded gastroplasty, 1.8% silicone band gastroplasty, and 1.8% truncal vagotomy) and that many subjects, 49 PWS patients, were lost to follow-up [103].

Instead, in a study of 24 adolescents, PWS compared to 72 non-PWS subjects matched for age, gender and BMI, all underwent a laparoscopic SG, a reduction in BMI of 15 kg/m^2^ at 1 year and 11 kg/m^2^ at 5 years, respectively, was observed, no different than the non-PWS group, in the absence of re-hospitalization for complications during follow-up, suggesting that SG could be an effective and relatively low-risk procedure in these subjects [102]. In a report of 3 male adolescents with PWS subjected to MGB, an effective weight reduction, a 79% excess weight loss two years after surgery, without need for revision surgery was observed [83]. Similarly, 3 Chinese subjects with PWS underwent bariatric surgery (2 SG and 1 MGB) and after a median follow-up of 33 months a mean weight loss and percentage of excessive weight loss at 2 years of 32.5 kg (24.9–38.3 kg) and of 63.2% (range 50.5–86.2%), respectively, were observed, without major complication [3]. Therefore bariatric surgery, however considering its implications and risks, can be a therapeutic strategy in case of severe obesity and failure of other weight loss interventions in the PWS subject.

## Conclusion

Obesity represents one of the main complications of PWS. It predisposes to the risk of developing T2DM, dyslipidemia and cardiovascular events, mostly at a young age. GH treatment along with lifestyle interventions represent a promising approach to prevent the development of obesity in these patients. However, when obesity occurs in adulthood, the use of metformin, GLP-1RAs and naltrexone-bupropion along with lifestyle interventions have been reported to have positive effects. Data coming from on-going trials are needed to open new therapeutical scenarios in the management of obesity in PWS.

## Abbreviations

PWS: Prader–Willi syndrome
BMI: Body mass index
GH: Growth hormone
MR: Magnetic resonance
AG: Acylated ghrelin
UAG: Unacylated ghrelin
NPY: Neuropeptide Y
SSA: Somatostatin analogue
PP: Pancreatic polypeptide
PYY: Peptide YY
GHD: Growth hormone deficiency
EE: Energy expenditure
T2DM: Type 2 diabetes mellitus
HOMA-IR: Homeostasis model assessment index
HbA1c: Glycosylated hemoglobin
OGTT: Oral glucose tolerance test
GLP-1RAs: Glucagon-like peptide-1 receptor agonists
DPP-4: Dipeptidyl peptidase-4
SGLT2: Sodium-glucose co-transporter-2
GLP-1: Glucagon-like peptide 1
HDL: High-density lipoprotein
LDL: Low-density lipoprotein
VFA: Visceral fat area
OSAS: Syndrome of sleep apnea
LC: Low carbohydrate
LF: Low fat
HQ: Hyperphagia Questionnaire
POMC: Pro-opiomelanocortin
α-MSH: α-Melanocyte-stimulating hormone
ARC: Arcuate nucleus
FDA: Food and Drug Administration
DHK: Dykens Hyperphagia Questionnaire
SG: Sleeve gastrectomy
MGB: Mini gastric bypass
